# Recent Progress in antibacterial hydrogel coatings for targeting biofilm to prevent orthopedic implant-associated infections

**DOI:** 10.3389/fmicb.2023.1343202

**Published:** 2023-12-22

**Authors:** Mengxuan Wang, Yawen Zheng, Chuqiang Yin, Shiyou Dai, Xiao Fan, Ying Jiang, Xuequan Liu, Junqiang Fang, Bingcheng Yi, Qihui Zhou, Ting Wang

**Affiliations:** ^1^Department of Orthopaedic Surgery, The Affiliated Hospital of Qingdao University, Qingdao, China; ^2^Department of Bone Joint and Sports Medicine, Qingdao Hospital, University of Health and Rehabilitation Sciences (Qingdao Municipal Hospital), Qingdao, China; ^3^Department of Anesthesiology, The Affiliated Hospital of Qingdao University, Qingdao, China; ^4^Shandong Key Laboratory of Carbohydrate Chemistry and Glycobiology, Shandong University, Qingdao, China; ^5^Qingdao Key Laboratory of Materials for Tissue Repair and Rehabilitation, School of Rehabilitation Sciences and Engineering, University of Health and Rehabilitation Sciences, Qingdao, China; ^6^Hubei Key Laboratory of Biomass Fibers and Eco-Dyeing and Finishing, Wuhan Textile University, Wuhan, China

**Keywords:** surface modification, antibacterial hydrogel, orthopedic implants, infection prevention, biofilm

## Abstract

The application of orthopedic implants for bone tissue reconstruction and functional restoration is crucial for patients with severe bone fractures and defects. However, the abiotic nature of orthopedic implants allows bacterial adhesion and colonization, leading to the formation of bacterial biofilms on the implant surface. This can result in implant failure and severe complications such as osteomyelitis and septic arthritis. The emergence of antibiotic-resistant bacteria and the limited efficacy of drugs against biofilms have increased the risk of orthopedic implant-associated infections (OIAI), necessitating the development of alternative therapeutics. In this regard, antibacterial hydrogels based on bacteria repelling, contact killing, drug delivery, or external assistance strategies have been extensively investigated for coating orthopedic implants through surface modification, offering a promising approach to target biofilm formation and prevent OIAI. This review provides an overview of recent advancements in the application of antibacterial hydrogel coatings for preventing OIAI by targeting biofilm formation. The topics covered include: (1) the mechanisms underlying OIAI occurrence and the role of biofilms in exacerbating OIAI development; (2) current strategies to impart anti-biofilm properties to hydrogel coatings and the mechanisms involved in treating OIAI. This article aims to summarize the progress in antibacterial hydrogel coatings for OIAI prevention, providing valuable insights and facilitating the development of prognostic markers for the design of effective antibacterial orthopedic implants.

## 1 Introduction

Orthopedic implants are widely used for non-union repair, long bone fracture fixation, joint replacement, and joint arthrodesis (Goodman et al., 2013; Zhou et al., 2016; Wang et al., 2021b,c). These implants can be classified into permanent and temporary orthopedic implants (Jin and Chu, 2019). Permanent implants encompass joint replacements, whereas temporary implants comprise plates, screws, pins, wires, and intramedullary nails (Park and Lakes, 2007). However, the abiotic nature of these implants and inadequate aseptic practices during surgery can lead to bacterial adhesion and colonization, causing both *in situ* and exogenous infections (Ercan et al., 2011). These infections trigger the host’s innate immune system, resulting in immediate inflammatory and antimicrobial responses and the production of various effector molecules, such as cytokines, chemokines, and antimicrobial proteins, to combat the invading bacteria (Jo, 2019). Unfortunately, severe orthopedic implant-associated infections (OIAI) can have devastating consequences and incur substantial costs (Darouiche, 2004; Gallo et al., 2014). These include impaired integration of the implant with surrounding tissues, leading to implant loosening (Costerton et al., 1999), the development of complications such as osteomyelitis, septic arthritis, or prosthetic joint infections (Ribeiro et al., 2012), and in severe cases, amputation and mortality (Ercan et al., 2011). Common approaches for OIAI treatment involve antibiotic therapy, revision, and debridement accompanied by long-term antimicrobial therapy through surgical procedures and administration of antibiotics (Moran et al., 2010), while the treatment effect is limited (Malahias et al., 2020). It is important to note that these methods are only effective in inhibiting bacterial growth or killing bacteria before biofilm formation. Once biofilm has formed on the implant surface, bacteria undergo significant metabolic changes, resulting in biofilm thickening and protection against the host immune response and antimicrobial agents (Ji et al., 2021; Kragh and Richter, 2022). This allows bacteria to proliferate in organized and structured communities, leading to nutrient deprivation and entry into a metabolically inactive dormant state (Bocci et al., 2018). Therefore, the development of a novel strategy to target biofilm formation is necessary for effective clinical prevention of OIAI.

Traditional strategies to prevent bacteria-induced clinical infections mainly include debridement, therapeutic cleansing, and administration of antimicrobials (Kaiser et al., 2021). However, systemic antibiotics often require high doses that can lead to side effects (e.g., cytotoxicity) and bacterial drug resistance (Uddin et al., 2021). To address infections caused by drug-resistant bacteria, various antimicrobial materials [such as hydrogels (Dou et al., 2023; Zhang D. et al., 2023), and surface coatings (Wei et al., 2022)], and antimicrobial molecules [such as antibacterial peptides (Costa et al., 2021) and amphiphiles (Ye et al., 2022)] have been developed. While progress has been made in post-infection treatment, interventions for biofilm prevention have been increasing dramatically (Connaughton et al., 2014). Inspired by the philosophy that prevention is better than cure, how to prevent the occurrence of bacterial infection effectively rather than post-infection treatment has become an important research hotspot nowadays. Recent developments greatly enrich our knowledge that bacteria can adhere to implant surfaces through forces like Van der Waals, Coulomb, and hydrogen bonding, it is crucial to develop strategies that interfere with bacteria-implant surface interactions for effective prevention of OIAI (Zimmerli, 2014). Surface modification with antimicrobial coatings has gained significant attention as it can improve the biocompatibility and control inflammation responses of implants (Kumar et al., 2021; Bohara and Suthakorn, 2022; Chen et al., 2023). Hydrogels, which mimic the three-dimensional network of polymer chains (Ahmed, 2015), have been used as biomimetic materials and drug delivery systems to enhance antibacterial performance and reduce drug resistance (Li and Mooney, 2016; Mei et al., 2022; Hao et al., 2022a,b). The surface modification of antibacterial hydrogels to prevent OIAI by targeting biofilm formation has become a focus of research. Besides drug loading platform, the hydrogel can also be designed as biosensor (Wang et al., 2021a) and drug evaluation system (Song T. et al., 2023), with great translational medicine application potential.

This review provides an overview of the research progress and clinical potential of surface-modification strategies using antibacterial hydrogel coatings to prevent OIAI. The review is divided into two main sections: (1) the mechanisms underlying the occurrence of OIAI and the role of biofilms in exacerbating OIAI development; and (2) current strategies to impart anti-biofilm properties to hydrogel coatings and the mechanisms involved in treating OIAI. Given the high severity of clinical infections associated with orthopedic implants, it is crucial to develop suitable antimicrobial materials that exhibit high-efficiency antibacterial effects, long-term efficacy, biocompatibility, and the ability to target bacterial biofilms for surface modification of orthopedic implants. Therefore, this review highlights recent advancements in selective and effective surface modification strategies using antibacterial hydrogels and provides insights into the design of antimicrobial orthopedic implants for effective infection prevention.

## 2 Orthopedic implant-associated infections

### 2.1 Occurrence mechanisms of OIAI

#### 2.1.1 Causes of OIAI

**Figure 1:**
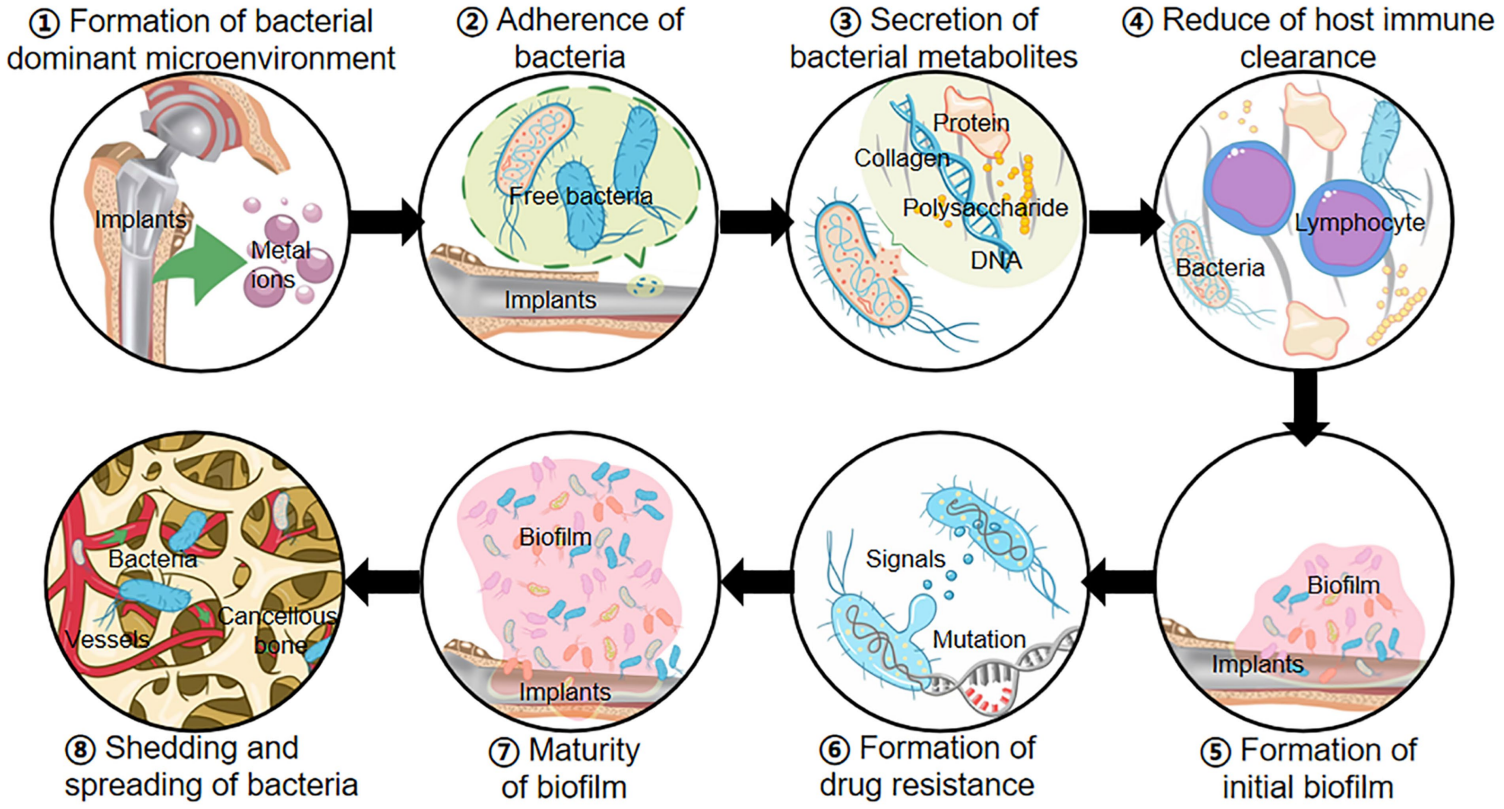
Occurrence mechanisms of OIAI: ① Implants release metal ions to form a bacterium-preferred environment; ② Surgical and blood-derived bacteria invade the wound and adhere to the surface of bone implants; ③ Bacteria secrete exopolysaccharides, nucleic acids, proteins, and other metabolites; ④ The accumulation of metabolites reduces the immune clearance of bacteria; ⑤ Bacterial biofilm initially forms on the implant surface; ⑥ Biofilm increases the drug resistance mutations in bacteria; ⑦ The formation of mature bacterial biofilms; ⑧ Bacteria in the biofilm shed and spread throughout the body in the bloodstream, causing OIAI.

With the development of the orthopedic medical device industry, the frequency of indwelling medical devices (e.g., implantable orthopedic medical devices) has greatly increased (Graf et al., 2019). Most orthopedic surgeries require implants, and the application of implants creates opportunities and increases the chances of OIAI (Figure 1; Darouiche, 2004). Traumatic bone tissue releases inducing factors such as magnesium ions to create a biofilm microenvironment (Kragh and Richter, 2022). When the sterile surgical environment is not guaranteed or the blood source of bacteria is transmitted to the surgical site, bacteria can secrete and accumulate various metabolic wastes such as nucleic acids, proteins, and extracellular polysaccharides, inhibit the immune function of host T/B lymphocytes, and promote the formation of biofilm on the surface of bone implants (Bocci et al., 2018). With further biofilm formation, the surface bacteria partially shed into the blood circulation, disseminated to various parts of the body and triggered OIAI (Masters et al., 2019). The formation of bacterial biofilm and hematogenous dissemination leads to poor efficacy of antibiotics and other drugs, persistent infection, and even the risk of secondary surgery to remove the implant (Zimmerli and Sendi, 2017). Table 1 is the current common classification of OIAI (Zimmerli, 2014), and based on the frequent and urgent medical background of OIAI, we will gradually explore the pathogenic causes and treatment strategies of OIAI and put forward our insights.

**Table 1:** The classification of orthopedic implant-associated infections (Zimmerli, 2014).

Orthopedic implant-associated infection	Common pathogenic bacteria	Classification	Clinical symptoms
Internal fixation-associated infection	Staphylococci, β-lactam-resistant gram-negative bacilli	Early postoperative infection (<3 weeks)	Erythema, local hyperthermia, protracted wound healing, and a secreting wet wound
		Delayed infections (3–10 weeks)	Persistent pain and/or signs of local inflammation, such as erythema, swelling, or intermittent drainage of pus
		Chronic infections(>10 weeks)	
Periprosthetic joint infections (PJIs)	*S. aureus* Coagulase-negative *staphylococci* *Streptococci*	Acute hematogenous	Sepsis, skin and soft-tissue infection, pneumonia, or enterocolitis
		Early postinterventional	Wound infection and pain
		Chronic	Chronic joint effusion, pain due to inflammation or implant loosening, local erythema, and hyperthermia, and occasionally by recurrent or permanent sinus tracts

Compared with the spine and trauma fields, elderly patients have a higher incidence of osteoarthritis, osteonecrosis of the femoral head, and other joint orthopedic diseases and face more application of bone implantation. Moreover, due to the age of the patients, the body’s resistance and tolerance are weaker than in young people (Leung et al., 2022), and the risk of postoperative OIAI is higher. Orthopedic surgery strictly requires the sterility of implants and environment. The positive pressure environment of the operating room can also greatly reduce the risk of bacterial contamination of the surgical site (Tarun et al., 2021). However, if the aseptic operation is not performed properly, bacteria will directly accumulate on the surface of the implant through the surgical wound or the air during the operation. Bacteria can be transmitted through the blood source from urinary tract infection, skin infection, and other infection routes, and enter the surgical site in the form of suspension, biofilm, or invasive cells (McConoughey et al., 2014). However, suspension and invasive bacteria are easily cleared by the host immune system or antibiotics and do not cause further infection, so bacterial biofilms become the main cause of OIAI (McConoughey et al., 2014; Davidson et al., 2019). The persistence and complications of bacterial infection after bone injury are also closely related to the formation and progression of bacterial biofilm (Krukiewicz et al., 2022). Therefore, the intractable nature of OIAI can be ascribed to the formation of bacterial biofilms on the surface of bone implants (Blanchette and Wenke, 2018).

#### 2.1.2 Currently developed strategies for combating OIAI

**Figure 2:**
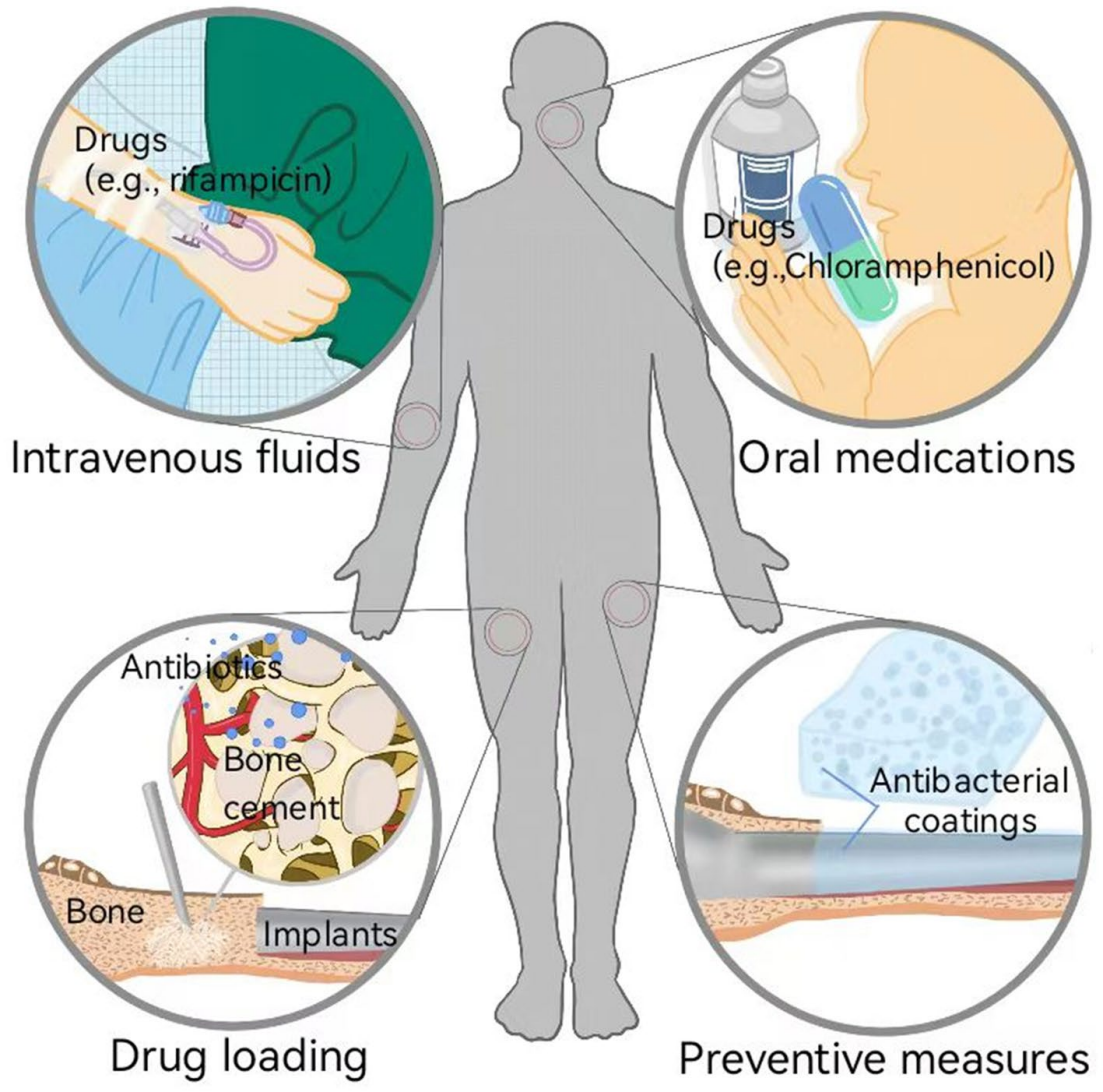
Currently developed strategies for combating OIAI: systemic therapies (e.g., intravenous fluids and oral medications of drugs) and local therapies (e.g., drug loading to inhibit bacteria growth and induce bacteria death, and preventive measures to inhibit bacterial adhesion and biofilm formation).

One crucial cause of OIAI is the susceptibility of traditional implant materials to bacterial biofilm adhesion and colonization on their surfaces (Gallo et al., 2014). Currently, systemic drug therapy is the primary treatment strategy for OIAIs (Figure 2; Inoue et al., 2017; Ishihama et al., 2021). Following surgical debridement, intravenous rifampicin is administered to rapidly control the bacterial load, followed by the application of orally administered drugs with improved bioavailability to inhibit systemic infection (Davidson et al., 2019; Guo et al., 2022). In cases of low systemic exposure, local drug therapy can be combined, and materials such as bone cement and calcium sulfate can be utilized as carriers for penicillin, cephalosporins, aminoglycosides, and quinolones to fill local bone defects (Lamret et al., 2020). These methods primarily focus on inhibiting bacterial growth or accelerating the death of free bacteria. However, the persistence and complications of bacterial infections after injured bone repair are closely associated with the formation and progression of bacterial biofilms (Krukiewicz et al., 2022). Once biofilms form on the implant surface, they become difficult to identify and eradicate, leading to hematogenous spread of infection caused by bacterial shedding and migration (Tsang et al., 2018). Moreover, severe infections resulting from bacterial biofilms often require implant removal through reoperation and may even involve the establishment of fistulas or amputation. This not only exacerbates physical trauma but also increases healthcare costs (Liu et al., 2019; Zhang et al., 2021). Consequently, the development of an effective therapeutic strategy to reduce bacterial adhesion and inhibit biofilm formation on bone implant surfaces, effectively decreasing OIAI incidence and improving the orthopedic surgery success rate, is a developing research trend in the antibacterial implant coatings field (Inoue et al., 2017; Ishihama et al., 2021).

### 2.2 Role of biofilm in aggravating the development of OIAI

#### 2.2.1 Definition, formation, and characteristics of biofilm

The concept of “biofilm” was first proposed by Bill Casterton in 1978 and was later defined in 2002 as a sessile community of microbial origin (Tarun et al., 2021). It has been confirmed that biofilm formation is a cooperative group behavior in which bacterial populations live embedded in a self-generated polymer extracellular matrix. This process is considered one of the most common cooperative behaviors exhibited by bacteria (Brao et al., 2021; Gao et al., 2023; Jiang et al., 2023; Liu et al., 2023). The biofilm primarily consists of sessile cell populations derived from microorganisms and quorum-sensing systems that serve as a cell–cell communication mechanism (Tsang et al., 2018). The quorum sensing (QS) systems synchronize gene expression in response to population cell density and coordinate the maturation, disassembly, and dispersion of the biofilm (Brao et al., 2021). Accumulating evidence demonstrates that bacteria within the biofilm can effectively grow while being protected from environmental stresses, such as immune system attacks and antimicrobial agents (Frieri et al., 2017). When nutrients or other resources become limited, biofilm dispersion occurs, allowing bacteria to escape and colonize new niches. Therefore, inhibiting biofilm formation is considered an effective strategy to prevent the exacerbated development of OIAIs (Qu et al., 2020). This necessitates a deep understanding of biofilm maturation progression and the unique characteristics of the formed biofilm (Brao et al., 2021).

**Figure 3:**
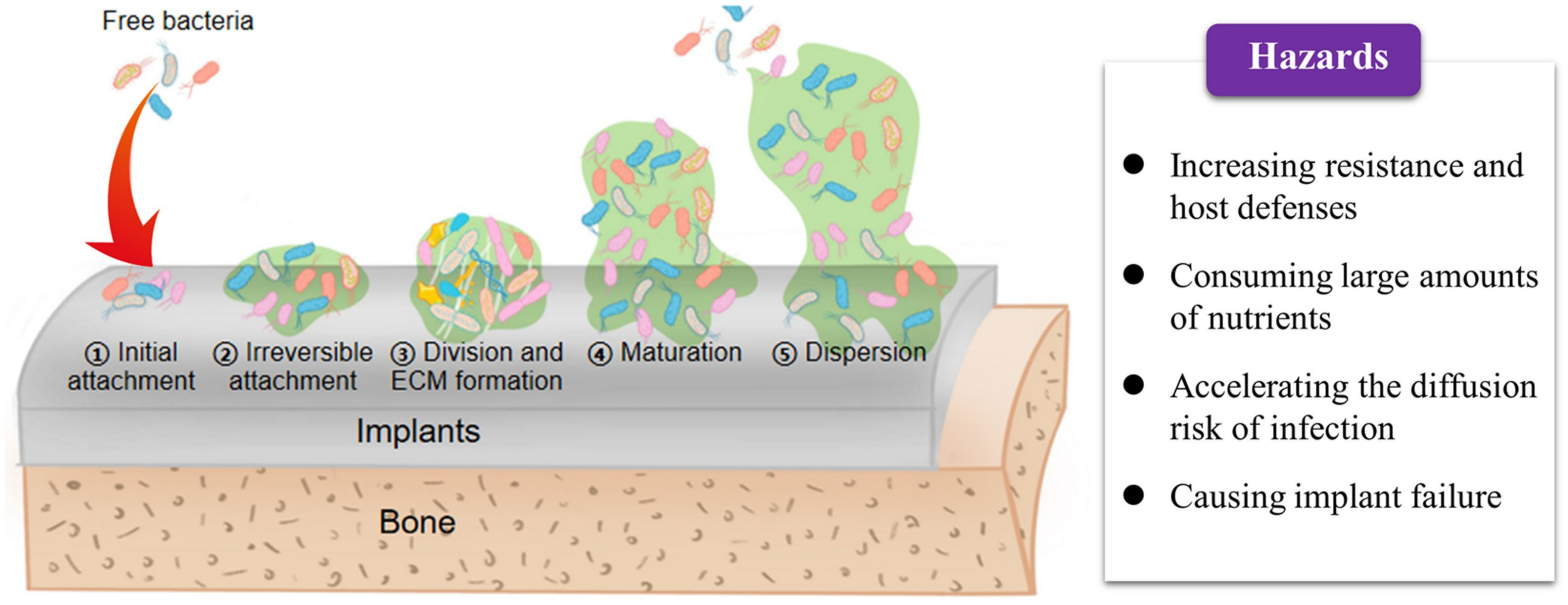
Biofilm formation process on the surface of orthopedic implants.

The entire process of biofilm formation on the surface of orthopedic implants primarily consists of three sequential stages (Figure 3): (1) irreversible adhesion to the surface, (2) bacterial division and extracellular matrix production, and (3) matrix disassembly and bacterial dispersion (Qu et al., 2020). The occurrence of OIAI is attributed to the abiotic surface of orthopedic implants, which provides an ideal interface for biofilm maturation (McConoughey et al., 2014; Davidson et al., 2019; Masters et al., 2019). Additionally, these implant surfaces exhibit poor immunosuppression and resistance to infection (Krukiewicz et al., 2022). Free bacteria can easily adhere to the implant surface through non-covalent bonds, such as electrostatic interactions and van der Waals forces, and accelerate biofilm formation by producing matrices such as water, extracellular polysaccharides, and extracellular DNA (Fany et al., 2017; Ghosh et al., 2020). If under unfavorable environmental conditions, bacterial autolysis, characterized by the lysis and release of extracellular DNA, can further enhance bacterial adhesion to the implant surface and stabilize the mature biofilm structure (Sabaté Brescó et al., 2017; Seebach and Kubatzky, 2019). Indeed, the initial adhesion step appears to be insufficient for the accumulation of quorum signals as bacteria are initially swimming freely in the media (Krukiewicz et al., 2022). However, as the attached bacteria divide and form microcolonies during the late stage, the increased population density improves quorum signals to sufficient levels, thereby coordinating the activation of biofilm maturation and disassembly (Qu et al., 2020).

After bacterial biofilm formation, changes in cell metabolism occur, either through chromosomal mutations or horizontal transfer of drug-resistance genes, to increase tolerance and resistance to antibiotics (Guo et al., 2022). This thickens the biofilms and further enhances bacterial resistance to host defenses and antibiotics (Donlan and Costerton, 2002). Moreover, the high cell density and closed matrix structure facilitate close communication between bacteria through chemical or electronic signals during biofilm maturation (Qu et al., 2020). This promotes horizontal gene transfer, sharing of virulence genes, and exchange of resistance genes (Masters et al., 2019). Such growth patterns can even give rise to the emergence of multi-drug-resistant bacteria (Frieri et al., 2017). Notably, the organized and structured bacterial communities within biofilms consume large amounts of nutrients at the periphery, resulting in central bacteria experiencing nutrient deprivation and entering a metabolically inactive dormant state (Bocci et al., 2018). Similarly, the dormant state of bacterial biofilms confers resistance to host immunity and inherent tolerance to antibiotics, leading to chronic infections and antibiotic overuse (Tsang et al., 2018; Lamret et al., 2020). Furthermore, bacteria trapped within mature biofilms can settle on new surfaces in search of new nutrient supplies, thereby promoting the spread and transfer of infection (Fany et al., 2017; Taha et al., 2018). For instance, osteomyelitis, a severe complication of bone implant infection, is closely associated with biofilm formation (Krukiewicz et al., 2022). During biofilm maturation, cell exfoliation leads to the detachment of bacterial debris, which can then attach to distant sites and contribute to the inflammatory response in bone infections (Mouzopoulos et al., 2011; Masters et al., 2019; Seebach and Kubatzky, 2019).

#### 2.2.2 Species of bacteria that hinder the curative effect of orthopedic implants

The bacteria associated with OIAI mainly include *Staphylococcus aureus*, *Enterococcus faecalis*, *Klebsiella pneumoniae*, *Acinetobacter baumannii*, and *Pseudomonas aeruginosa*, which can be categorized into two groups: Gram-positive and Gram-negative bacteria (Liu et al., 2019). Due to significant differences in structure and properties, these two groups of bacteria exhibit varying levels of pathogenicity during the OIAI process (Liu et al., 2019). For instance, *Staphylococcus aureus*, a highly virulent Gram-positive bacterium (Graf et al., 2019), is the primary pathogen responsible for OIAI (Gatti et al., 2022), accounting for approximately one-third of bone implant infections, including artificial joints and bone fixation devices (Muthukrishnan et al., 2019). With increasing multidrug resistance, *Staphylococcus aureus* has become more resistant to antibiotics and can cause persistent chronic infections in tissues and organs. Previous studies have demonstrated the high adaptability of *Staphylococcus aureus* to the human environment (Arciola et al., 2018). Additionally, it employs alternative resistance mechanisms, such as the formation of dormant subpopulations and complex biological communities, to evade the host immune system and environmental stressors (Flemming and Wingender, 2010; Guo et al., 2022), thereby leading to severe local infections or osteomyelitis (Masters et al., 2019; Muthukrishnan et al., 2019; Guo et al., 2022).

For the mechanisms involved in bacteria that hinder the curative effect of orthopedic implants, accumulating evidence suggests that when orthopedic implants become contaminated with bacteria, the adhered bacteria tend to activate the host’s immune system (Costerton et al., 1999). This activation leads to the recruitment of immune cells to the site of infection, which in turn release pro-inflammatory factors, creating an inflammatory microenvironment to combat the bacteria (Arciola et al., 2018). However, as bacteria grow and form biofilms on the implant surface, they become resistant to engulfment by immune cells (Guo et al., 2022). These bacteria not only continue to stimulate immune cells to produce more pro-inflammatory mediators but also activate macrophages and neutrophils to release cytotoxic substances that damage surrounding tissues (Qu et al., 2020). Consequently, the sustained local inflammatory response to infection disrupts the homeostasis of osteogenesis, resulting in bone resorption and leading to tissue degradation and osteolysis (Krukiewicz et al., 2022). Moreover, OIAI interferes with the host’s bone healing process, perpetuating bone resorption and ultimately resulting in septic loosening of bone implants and transplant failure (Bocci et al., 2018).

#### 2.2.3 Main mechanisms involved in inhibiting/destroying biofilm for OIAI treatment

The formation of bacterial biofilms is a dynamic process that can be divided into several stages, including reversible bacterial adhesion and colonization, irreversible bacterial adhesion and colonization, biofilm maturation, and bacterial shedding and re-adhesion (Kragh and Richter, 2022). The initial adhesion of free bacteria to the surface of bone implant materials is crucial for initiating biofilm formation (Costerton et al., 1999), which is influenced by various factors, including the properties of the implant material surface, bacterial properties, and other factors (McConoughey et al., 2014). Once free bacteria adhere to the surface of orthopedic implants, they secrete quorum-sensing signaling molecules to monitor changes in the surrounding environment, regulate the expression of related genes, and produce a significant amount of extracellular polymers to fill the intercellular space and enhance mechanical strength (Subramani and Jayaprakashvel, 2019). The presence of such a QS system is not only crucial for biofilm formation but also for drug resistance and pathogenicity (Inoue et al., 2017).

In the context of OIAI treatment, inhibition of QS therefore is considered one of the main mechanisms by which orthopedic implants inhibit or destroy biofilms. This involves blocking bacterial communication signals to prevent bacterial aggregation and biofilm formation (Subramani and Jayaprakashvel, 2019). Additionally, inhibiting bacterial adhesion and colonization on the implant surface is also a critical mechanism for OIAI treatment, as bacterial adhesion is a key event preceding biofilm formation. For instance, antimicrobial peptides (AMPs) or other substances can act on both Gram-positive and Gram-negative bacteria to inhibit the adhesion process by promoting fimbria synthesis, interfering with QS between bacteria, clearing and destroying extracellular polymers between bacteria, and penetrating the lipid bilayer for sterilization using their amphiphilic properties (Ghosh et al., 2020). As such, modifying the surface of implants with antibacterial substances is considered an effective strategy for OIAI treatment (Xv et al., 2019; Ishihama et al., 2021). Directly killing bacteria is another effective approach to inhibit biofilm formation. For example, non-biodegradable nano-drug delivery systems can disrupt the normal structure of bacteria (Ercan et al., 2011; Slavin et al., 2017), while lipid nano-systems can fuse with bacterial cell membranes to release drugs into the bacteria (Sani and Separovic, 2016), both playing important roles in OIAI treatment. Once the biofilm has reached the mature or later stages, the destruction of the tight extracellular polymer structure between bacteria using drugs (e.g., dispersants or deoxyribonucleases) can be considered another critical mechanism in destroying established biofilms (Ghosh et al., 2020). To reduce biofilm resistance and increase the likelihood of biofilm destruction, metabolic promoters can be used as a combined mechanism to awaken dormant bacteria in biofilms and inhibit drug efflux pumps on bacterial biofilms (Bocci et al., 2018; Graf et al., 2019).

## 3 Development of antibacterial hydrogel coating to prevent OIAI by inhibiting biofilm formation

**Figure 4:**
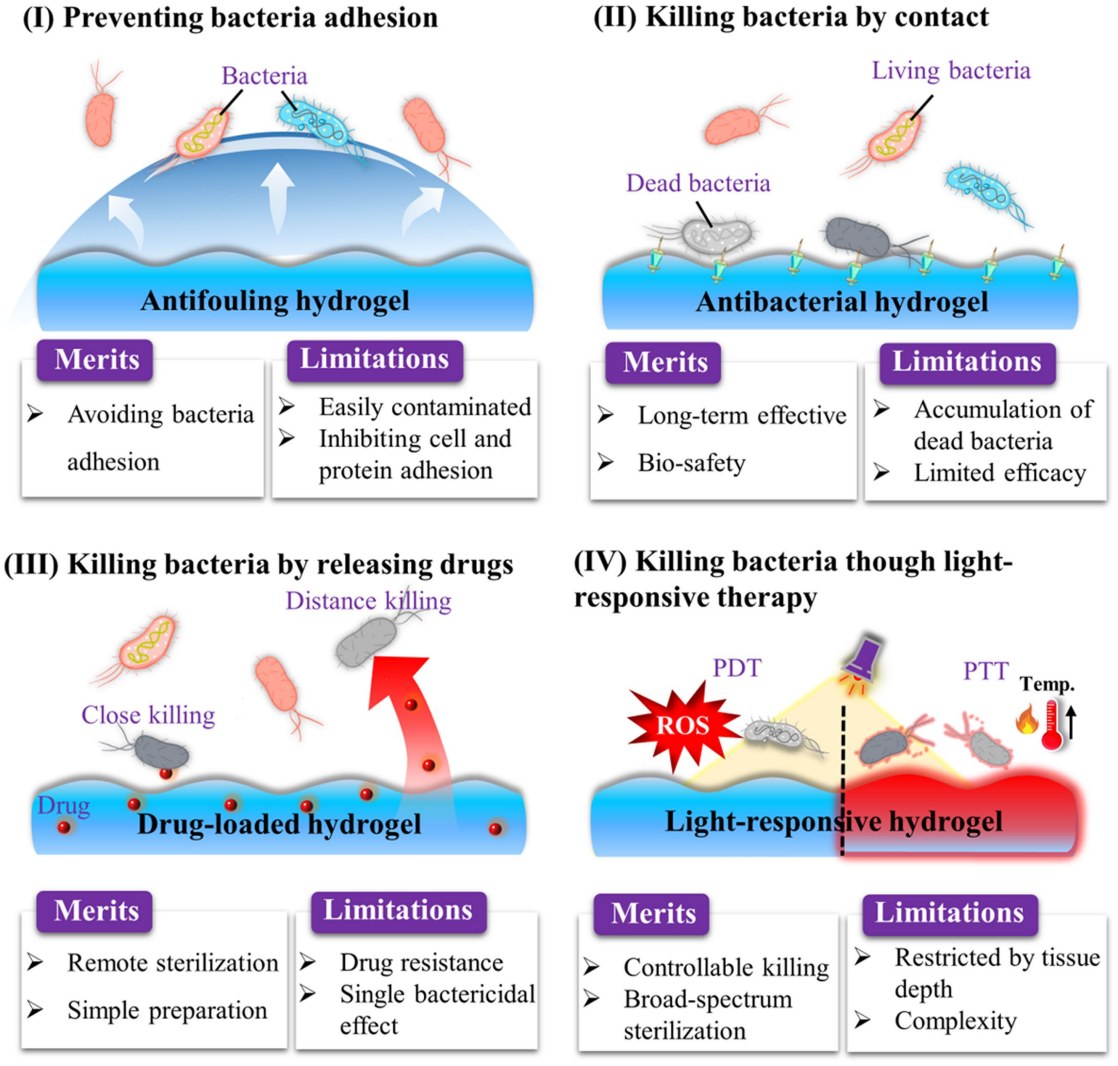
Strategies to endow hydrogel coating with anti-biofilm properties for OIAI prevention.

The biofilm formation process contains irreversible adhesion, extracellular matrix synthesis, colony formation, and maturation (Erathodiyil et al., 2020). Thus, to inhibit OIAI development, it is necessary to modify orthopedic implants with antibacterial materials to prevent bacterial attachment and biofilm formation. To date, various types of antibacterial hydrogels have been investigated, including those based on antifouling polymers (Erathodiyil et al., 2020), AMPs (Lin et al., 2020), cationic materials (Zhao et al., 2022), and photodynamic/photothermal agents (Ma et al., 2022; Figure 4). In this section, we primarily focus on the development of strategies for endowing hydrogel coating with anti-biofilm properties for OIAI prevention.

### 3.1 Construction of bacteria-repelling hydrogel coating

**Figure 5:**
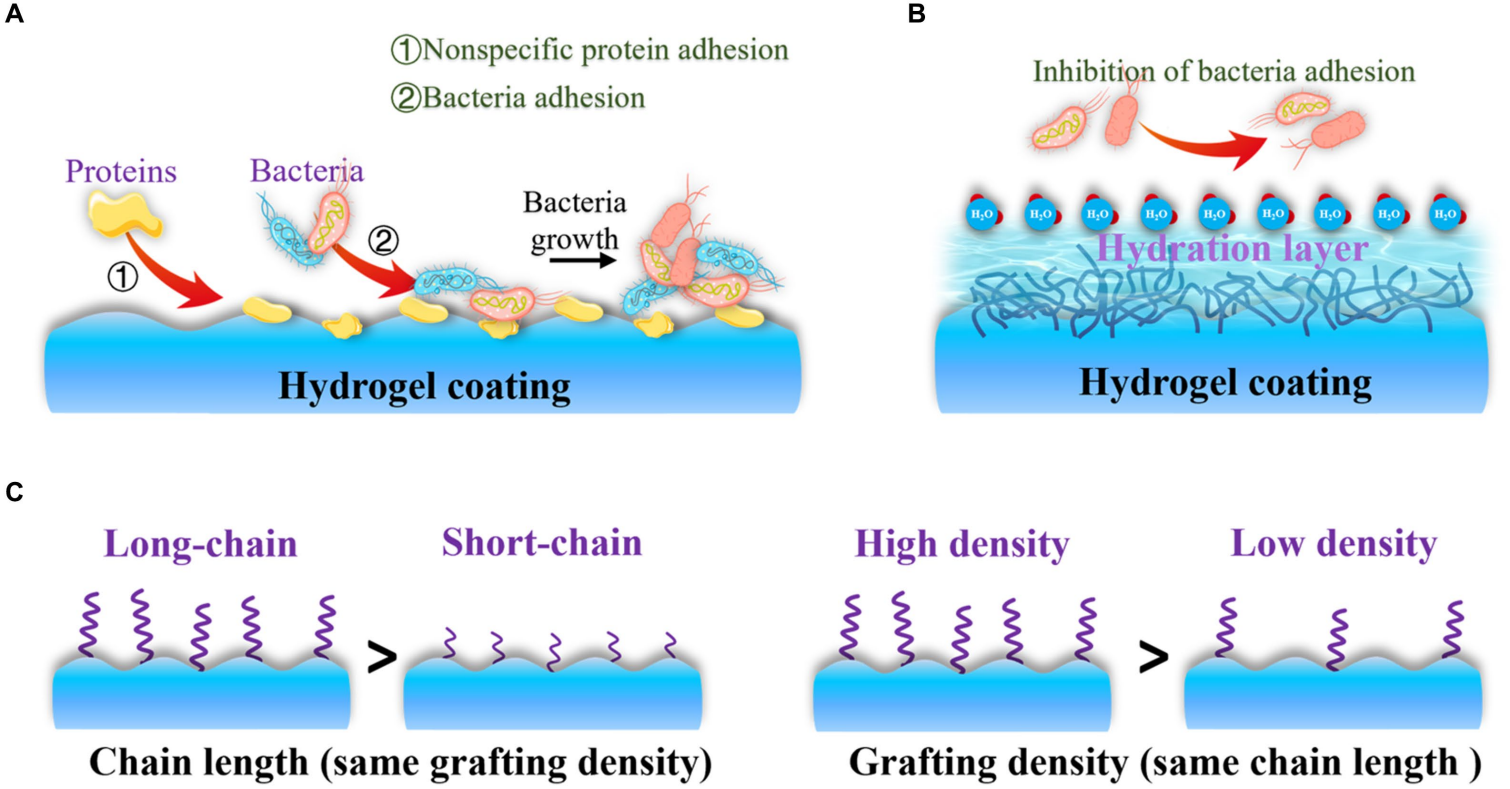
Antifouling and bacteria-repelling hydrogel surfaces: **(A)** Nonspecific protein adhesion promotes bacterial adhesion; **(B)** Antifouling surfaces create a tightly bound hydration layer at the interface to inhibit proteins and bacteria adhesion; **(C)** Bacteria-repelling property is influenced by chain length and grafting density of antifouling molecules.

After drug-resistant bacteria attach to the surface of orthopedic implants, the failure of antibiotic treatment often leads to the formation of dangerous bacterial biofilms on the implant surface (Zhu et al., 2022). This situation creates an urgent need to develop effective strategies to enhance the antibacterial properties of orthopedic implants. Recent evidence has shown that nonspecific protein adsorption, which is a recognized challenge for orthopedic implants, can interact with the cell surface of bacteria and initiate downstream bacterial adhesion-related signaling pathways (Figure 5A; Erathodiyil et al., 2020). Once bacterial adhesion is complete, bacteria mature, form microbial colonies, and generate biofilms on the implant surface. This means that bacteria can adhere to the surface due to nonspecific protein adsorption, regardless of whether the implant surface is modified with functional molecules or not. To address this problem, extensive efforts have been made to develop antifouling and bacteria-repelling hydrogel surfaces using various hydrophilic polymers, including poly (ethylene glycol) (PEG)-based (Lundberg et al., 2010), zwitterionic polymer-based (Leigh et al., 2019), and poly(N-vinylpyrrolidone)-based (Guo et al., 2019) hydrogels. These hydrogels create a tightly bound hydration layer at the interface, which acts as a physical barrier that proteins and bacteria find thermodynamically unfavorable to overcome (Figure 5B). As a result, non-specific protein adsorption and bacterial adhesion on coated implants are inhibited (Chen et al., 2021).

The bacteria-repelling property is primarily influenced by parameters such as chain length (Chen et al., 2019) and grafting density of antifouling molecules (Sun et al., 2014). Therefore, controlling the chain segments allows for optimal inhibition of bacteria adhesion (Figure 5C). In addition to their antifouling properties, surface modifications driven by polyphenols have recently garnered attention for site-specific and prolonged elimination of bacteria adhesion and OIAI. This is due to the active functional groups present in polyphenols, such as dihydroxyphenyl and trihydroxyphenyl, which can also act as natural reducing/exfoliating agents (Zhou et al., 2020). Incorporating various bioactive molecules into advanced implants can be achieved using these functional groups. For instance, tannic acid exhibits notable antiviral, antibacterial, and antioxidant properties due to its pyrogallol and catechol groups (Kaczmarek-Szczepańska et al., 2023), which can interact with biomolecules and metal ions in bacteria. This interaction increases cell membrane permeability and disrupts cell membrane stability. The excessive phenolic functional groups of tannic acid also offer good coordination sites for the post-modification of bone-inducing biomolecules, promoting biocompatible bone adhesives and guiding bone regeneration (Bai et al., 2020). Similarly, polydopamine-modified hydrogels have gained significant interest for improving the bacteria-repelling ability of orthopedic implants. Additionally, polydopamine can act as a reductant to introduce metal ions, thereby enhancing bone regeneration (Xia et al., 2022; Shen et al., 2024).

### 3.2 Endowing hydrogel coatings with contact-killing property

**Figure 6:**
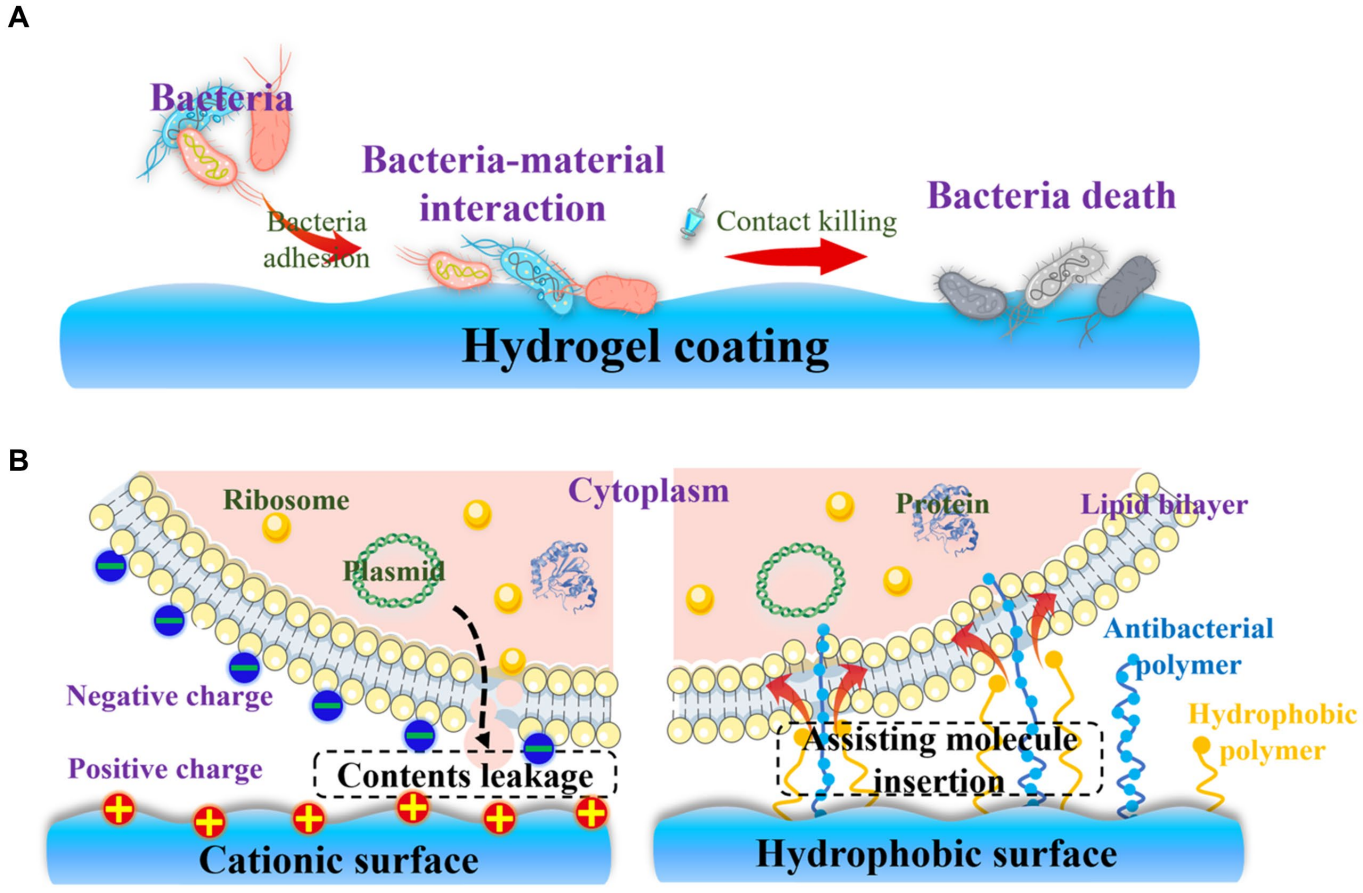
Contact-killing hydrogel surfaces: **(A)** Strategies for the contact-killing property; **(B)** Mechanisms involved in the contact-killing behavior of cationic and hydrophobic surfaces.

Biofilm formation necessitates the initial adhesion of bacteria to the surface of orthopedic implants. Consequently, developing an effective strategy to eradicate bacteria after adhesion is considered another crucial step in preventing OIAI. Contact-killing hydrogel coatings, which involve the irreversible anchoring of bactericidal agents onto the surface through non-leaching mechanisms, present a promising solution to this problem (Figure 6A). In contrast to the passive action of bacteria-repelling hydrogels, these bactericidal coatings actively work to eliminate bacteria upon their adhesion. The contact-killing behavior of these coatings is attributed to their ability to disrupt bacterial membranes and inhibit bacterial growth, ultimately leading to the death of pathogens (Costerton et al., 1999; Hao et al., 2022a,b). Considering the negative charge of bacteria, the commonly used biomaterials for contact-killing hydrogel coatings typically incorporate cationic groups and hydrophobic moieties (Lu et al., 2015). This choice is based on two main factors (Figure 6B): cationic surfaces can capture bacteria and disrupt the integrity of the bacterial membrane through electrostatic interactions, leading to leakage of cellular contents and subsequent bacterial death (Tischer et al., 2012); and hydrophobic surfaces facilitate the insertion of antibacterial molecules into the lipid composition of the bacterial membrane (Kopiasz et al., 2021). The widely investigated biocides that impart contact-active bactericidal activity to hydrogel surfaces include quaternary ammonium compounds (QACs) and AMPs.

QACs are highly efficient in exhibiting contact-killing activity against both Gram-positive and Gram-negative bacteria due to their typical structure, featuring four alkyl groups covalently attached to a central nitrogen atom. The cationic charge of QACs contributes to their bactericidal properties, while the hydrophobicity of the long alkyl chains also exhibits excellent antibacterial activity. Incorporating QACs into hydrogels has been identified as a simple and effective approach to confer bactericidal characteristics to implants, with an almost 100% killing efficiency against *E. coli.* (Li et al., 2009). However, this killing effect is non-specific.

AMPs, which serve as the first line of defense against invading pathogens in various organisms, display strong contact-active bactericidal activity against a broad spectrum of bacteria, including drug-resistant strains (Zhang and Gallo, 2016). They exert an antibacterial effect mainly through membrane disruption, macromolecule synthesis inhibition, and intracellular dysfunction (Michael and Nannette, 2003). This activity is attributed to their abundant cationic charge derived from arginine and lysine residues, as well as a high proportion of hydrophobic amino acids. The improved antibacterial activity of AMPs is further enhanced by their secondary structures resulting from their amphipathic features (Bahar and Ren, 2013). Additionally, AMPs can enter bacteria and interfere with nucleic acid metabolism (Batoni et al., 2016). Compared to traditional antibiotics, AMPs exhibit strong antibacterial activity against a wide range of microorganisms, including Gram-positive and Gram-negative bacteria, fungi, and even viruses (Aoki et al., 2012). Thus, they are considered promising alternatives to antibiotics. To construct a contact-killing surface, AMPs can be covalently reacted with the hydrogel network (Zhu et al., 2019) or self-assembled into physically crosslinked hydrogels possibly for surface modification of orthopedic implants (Guo et al., 2021). However, the presence of nonspecific protein absorption on the implant surface may block the active moieties of these antibacterial materials, thereby impairing their antibacterial efficacy (Wang et al., 2016, 2017). Additionally, the accumulation of dead cell and autolytic bacteria on the implant surface can assist in the formation of biofilms (Arciola et al., 2018). Therefore, for optimal coating performance, the contact-killing strategy is often combined with other approaches, such as the bacteria-repellent method.

### 3.3 Development of hydrogel-assisted delivery of non-antibiotic therapeutics

**Figure 7:**
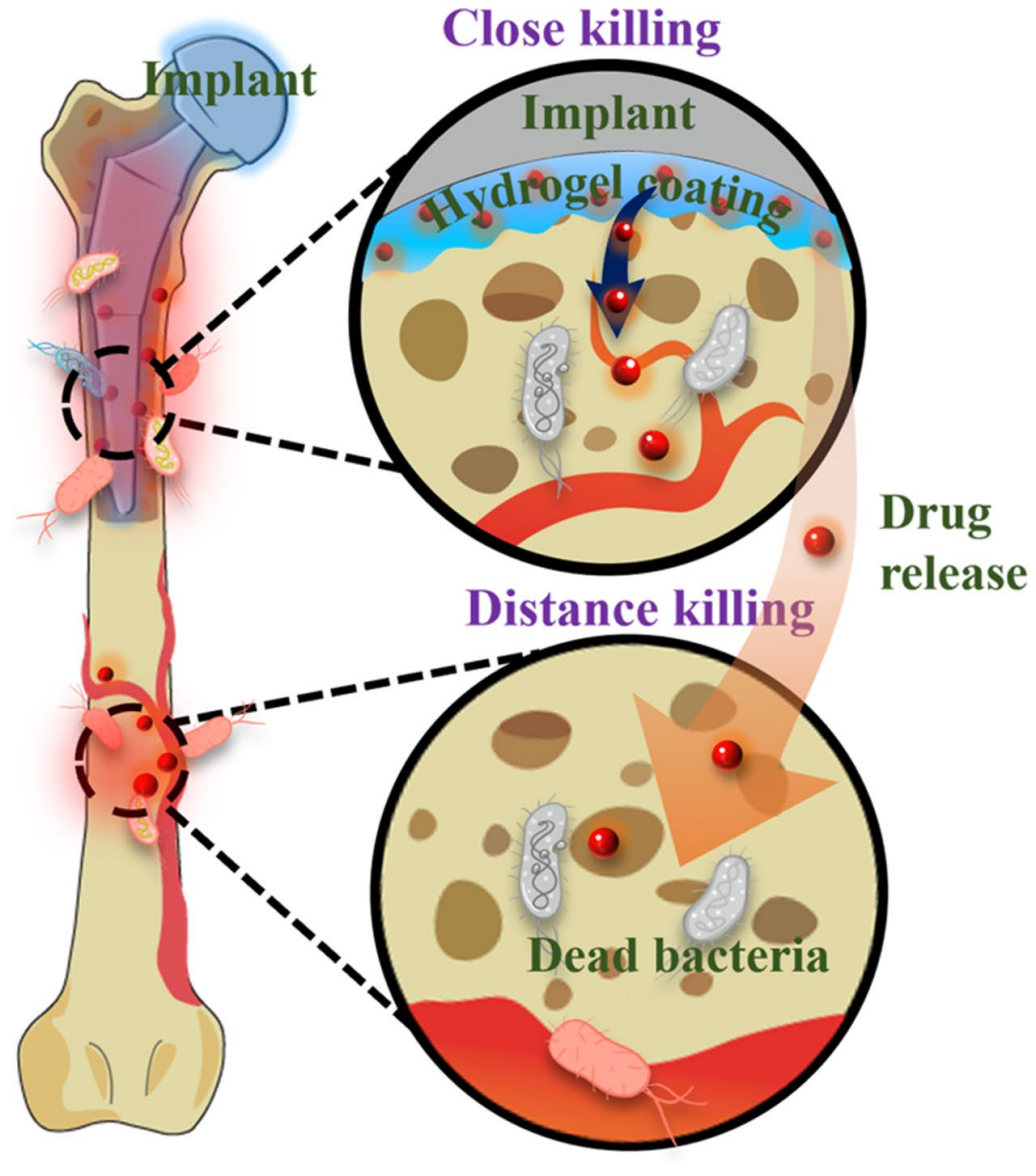
Schematic diagram of hydrogel-assisted drug delivery for OIAI treatment.

The emergence of antibiotic-resistant bacteria and the limited efficacy of antibiotics against biofilms have highlighted the need for the development of biomaterial-assisted delivery of non-antibiotic therapeutics for the treatment of OIAI. Hydrogel, a water-rich polymer network, offers remarkable versatility in encapsulating and delivering drugs (Dreiss, 2020; Yang, 2022). Novel hydrogel systems have been developed to load various biocides, including metal nanoparticles and AMPs. These systems enable controlled delivery of antimicrobial agents, reducing potential systemic adverse effects, while also achieving sustained release at the infection site to enhance the treatment of antibiotic-resistant and recurring infections. Unlike bacteria-repelling coatings, such release-killing surfaces may allow initial bacterial attachment (Figure 7). However, the diffusion of antimicrobial agents from these surfaces kills the attached bacteria and even eliminates surrounding bacteria in adjacent tissues. Nonetheless, it is crucial to ensure sufficient loading capacity and localized release of antimicrobials to effectively mitigate bacterial infections and avoid systemic adverse effects associated with antimicrobial coatings.

Metal materials, such as silver, copper, and zinc oxide (Leng et al., 2021) et al., have garnered significant attention due to their unique optical and electronic properties, as well as their excellent antimicrobial activity (Zhou et al., 2017, 2018; Liu et al., 2020; Yu et al., 2020; Yang et al., 2021). For example, silver nanoparticles exert their bactericidal effects by damaging the bacterial outer membrane and generating reactive oxygen species, which leads to oxidative damage to cellular structures. Silver nanoparticles release Ag^+^ ions that interact with the bacterial cell membrane, causing rupture and subsequent leakage of cellular contents. The released Ag^+^ ions can also penetrate bacterial cells and inhibit DNA replication. In addition to metal nanoparticles, AMPs are commonly incorporated into the network structure of hydrogel coatings. For instance, the antimicrobial peptide KRWWKWWRR (HHC-36) and osteoconductive silicate nanoparticles were encapsulated into gelatin methacryloyl hydrogel coatings modified with catechol motifs to prevent biofilm formation and promote osteogenesis on the surface of titanium implants (Cheng et al., 2017). Despite the success achieved in hydrogel-assisted delivery of non-antibiotic therapeutics, the antimicrobial activity of these systems is often short-term, and the burst release of antibacterial agents can lead to adverse side effects. Hence, the development of stimuli-responsive hydrogel coatings that enable sustainable and on-demand release of antimicrobial agents is necessary to mitigate adverse effects and premature depletion of the drug reservoir. In response to multiple responses, the hydrogel system can be suitable for more application situations (Li et al., 2022). Furthermore, the integration of two or more different agents in hydrogel-assisted delivery can be designed to produce synergistic effects, thereby enabling diverse functionalities for the prevention of OIAI and promotion of osteogenesis.

### 3.4 Endowing hydrogel coating with photodynamic or photothermal conversion effect

**Figure 8:**
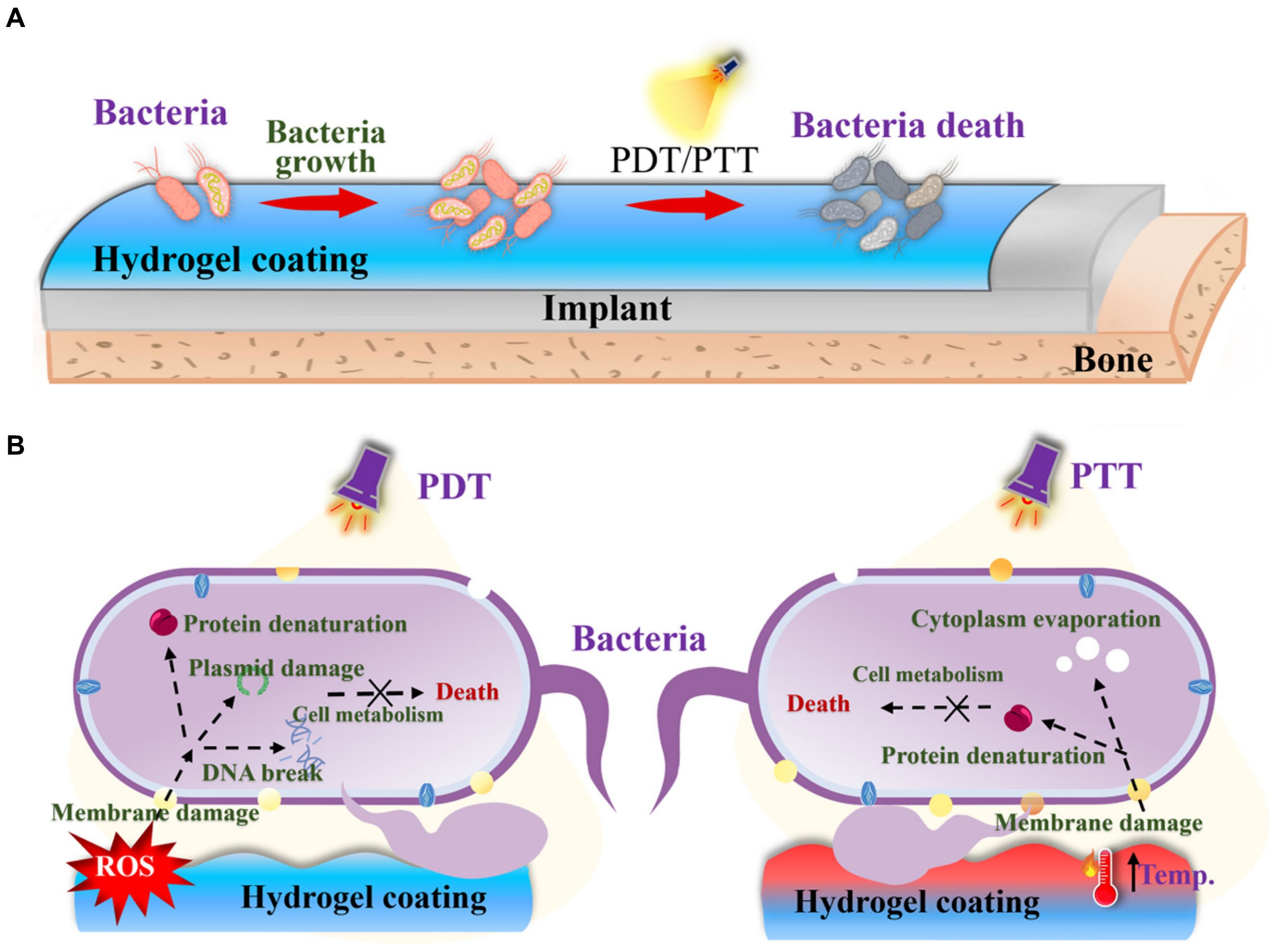
**(A)** Schematic diagram of PDT and PTT strategies for OIAI treatment. **(B)** PDT and PTT bactericidal mechanism.

Though many contact or release-killing surfaces have been reported to inhibit bacterial growth on orthopedic implants, their limited efficacy in preventing biofilm formation and potential adverse side effects of drugs necessitates the exploration of alternative therapeutic strategies. Furthermore, the presence of dead bacteria on the surface triggers undesirable immune and inflammatory responses. Photodynamic therapy (PDT) and photothermal therapy (PTT) emerge as promising approaches (Figure 8), especially PTT for minimally invasive nature, non-surgical application, and deep tissue penetration (Chen et al., 2020; Song et al., 2022; Song W. et al., 2023). Many metal oxides possess the phototherapy ability (Wu et al., 2022; Zhang W. et al., 2023), and can play a role as enzymes to perform two functions (Wang et al., 2021; Shen et al., 2023). Meanwhile, this system can specifically respond to an inflammatory environment (Cui et al., 2023). These systems achieve anti-biofilm and anti-inflammatory effects through photothermal therapy, combined with antioxidant activity, or recyclable reactive oxygen species scavenging ability (Cui et al., 2023; Ding et al., 2023). For example, antibacterial PDT utilizes laser irradiation and molecular oxygen to damage bacteria, including multidrug-resistant strains (Jia et al., 2019). This approach can be applied without causing immunosuppressive or myelosuppressive effects (Misra et al., 2010). Accumulating evidence supports the efficacy of PDT in killing bacteria through the production of singlet oxygen (^1^O_2_) and the generation of other reactive oxygen species (Misba et al., 2018). PTT has also garnered significant attention as a potent tool against bacterial infections due to its broad-spectrum bactericidal effect, in contrast with chemotherapy. The induced heat by photothermal agents leads to bacterial protein denaturation and cell death (Wickramasinghe et al., 2020). Various functional materials, including polydopamine (Yuan et al., 2019), have been utilized as photothermal agents to combat bacterial infections. Interestingly, some microalgae as a biomaterial can also function in the PDT field (Zhong et al., 2021). And some functional organic dyes are multifunctional materials, which can perform various functions including phototherapy (Cheng et al., 2020).

Overall, each strategy offers distinct advantages and limitations. The selection of an appropriate coating to modify implants should be based on clinical requirements. For instance, the bacteria-repelling strategy may not be suitable for patients with chronic diseases due to its limited bioactivity. Likewise, light-responsive therapy may be constrained in the applications in deep tissue. Furthermore, to achieve optimal results, a combination of both strategies can be employed to achieve synergistic bactericidal efficacy.

## 4 Conclusion and future perspectives

Because of the abiotic nature of orthopedic implants that allows bacterial adhesion and biofilm formation on the implant surface, orthopedic implant-associated infections have been developed as a significant concern in clinical practice. With the emergence of antibiotic-resistant bacteria and the limited efficacy of drugs, alternative therapeutics based on antibacterial hydrogels have been extensively investigated to coat orthopedic implants, offering a promising approach to target biofilm formation and prevent OIAI. To date, numerous antibacterial hydrogel coatings involved in bacteria-repelling properties, contact-killing ability, hydrogel-assisted delivery system, or photodynamic/photothermal conversion effect have been reported, providing powerful tools for combating bacterial infections. Hence, this review focuses on providing an overview of recent advancements in the application of these antibacterial hydrogel coatings for preventing OIAI by targeting biofilm formation.

Although great advancements in antibacterial hydrogel coatings have been achieved in preventing OIAI, these strategies generally neglect the influence of microenvironment changes (e.g., inflammatory response), which need to be considered in future design of antibacterial hydrogel coatings. Besides, as the aging population and increased bacterial drug resistance contribute to an increased incidence of inflection-related diseases, developing a novel anti-infection strategy to design effective antibacterial hydrogel coatings are highly desirable to treat OIAI and mitigate severe complication. Additionally, in the future development of hydrogel coatings, emphasis should be placed on the integration of multiple functions, such as promoting osteogenesis, inhibiting scar formation, and inducing angiogenesis. To note, the application of hydrogel coating often restricts the direct integration of implants with tissue, while the gradual degradation of hydrogel can impede the interface conjunction necessary for bone healing. Therefore, the design of an antibacterial hydrogel coating with the ability to promote bone healing and reconstruction is speculated. Alternatively, a more effective approach would be to directly incorporate antibacterial properties into the implant rather than relying on the application of an antibacterial hydrogel coating. Considering the presence of cytotoxicity and non-degradability in the currently designed hydrogel coatings, optimizing the biological characteristics of hydrogel systems can further improve the efficacy of these local administration systems.

## Author contributions

MW: Writing – original draft, Writing – review & editing, Investigation, Methodology, Software. YZ: Investigation, Methodology, Software, Writing – original draft, Writing – review & editing. CY: Conceptualization, Methodology, Supervision, Writing – original draft, Writing – review & editing. SD: Formal analysis, Validation, Writing – review & editing. XF: Formal analysis, Validation, Writing – review & editing. YJ: Software, Writing – review & editing. XL: Methodology, Validation, Writing – review & editing. JF: Methodology, Validation, Writing – review & editing. BY: Funding acquisition, Methodology, Project administration, Resources, Supervision, Validation, Writing – review & editing. QZ: Funding acquisition, Methodology, Project administration, Resources, Supervision, Validation, Writing – review & editing. TW: Funding acquisition, Methodology, Project administration, Resources, Supervision, Validation, Writing – review & editing.
